# Mobile phone filmmaking in health promotion. Addressing problematic social media use in Swedish youth

**DOI:** 10.1093/eurpub/ckac129.421

**Published:** 2022-10-25

**Authors:** H Dahlqvist, K Gillander Gådin

**Affiliations:** Mid Sweden University, Sundsvall, Sweden

## Abstract

**Background:**

Digital media is an important part in the everyday lives of young people. However, hate, threats, and harassment on social media is becoming a global public mental health issue among youth. To promote mental health in this age group, this issue needs to be addressed, and community-based health promotion, in particular participatory interventions, are needed to make measures taken meaningful for youth. The aim of the study was twofold; to teach participatory mobile phone filmmaking to Youth Community Center (YCC) staff; and to investigate how they assess this method regarding feasibility, relevance, costs, resources needed, and time considerations.

**Methods:**

YCC staff participated in a two-day mobile phone filmmaking training. Group interviews that were recorded and transcribed verbatim were conducted and field notes were taken. Data was deductively analyzed in accordance with Elo and Kyngäs (2008).

**Results:**

YCC staff found the technology was easily accessible and the method needs limited extra resources. It has the potential to promote creativity and can be used as a means to involve young people in describing the issue in their own words, and to find a solution to the issue at hand. The method also has the potential to reach and engage the whole community as organizing a mobile phone film event is part of the process. Time consumption was not perceived as an issue, as staff is free to plan activities as they see fit.

**Conclusions:**

According to YCC staff, participatory mobile phone filmmaking has the potential to promote positive interactions on social media among youth. This in turn has the potential to promote wellbeing of young people. Recommendations are that this method is tested among young people and investigate if it is a helpful intervention to promote mental health in this age group.

**Key messages:**

• Participatory mobile phone filmmaking may be useful in youth mental health promotion.

• The method is cheap, easily accessible, and have the potential to involve the whole community.

